# InAs/GaAs quantum dots with wide-range tunable densities by simply varying V/III ratio using metal-organic chemical vapor deposition

**DOI:** 10.1186/1556-276X-8-367

**Published:** 2013-08-28

**Authors:** Senlin Li, Qingqing Chen, Shichuang Sun, Yulian Li, Qiangzhong Zhu, Juntao Li, Xuehua Wang, Junbo Han, Junpei Zhang, Changqing Chen, Yanyan Fang

**Affiliations:** 1 Wuhan National Laboratory for Optoelectronics, School of Optical and Electronic Information, Huazhong University of Science and Technology, Wuhan 430074, People’s Republic of China; 2 State Key Laboratory of Optical Materials and Technologies, School of Physics and Engineering, Sun Yat-Sen University, Guangzhou 510275, People’s Republic of China; 3 Wuhan National High Magnetic Field Center, Huazhong University of Science and Technology, Wuhan 430074, People’s Republic of China

**Keywords:** InAs quantum dots, V/III ratio, MOCVD

## Abstract

The complicated behaviors of InAs/GaAs quantum dots with increasing V/III ratio associated with several competing mechanisms have been described. The results demonstrate that the densities of InAs quantum dots can be tuned in a wide range from 10^5^ to 10^10^ cm^−2^ by simply manipulating V/III ratio via metal-organic chemical vapor deposition. These results are mainly ascribed to the changes of coverage and In adatom migration length due to the increasing V/III ratio.

## Background

Self-assembled InAs/GaAs quantum dots (QDs) have been widely investigated due to their applications in a variety of optoelectronic devices. High-density QD-based structures are usually needed for devices like lasers and solar cells [1-5], while low-density QD-based structures are preferred for devices such as single-photon sources [6]. Due to the great effects of growth kinetics on QDs’ density and size, both high- and low-density QDs may be acquired by choosing suitable growth techniques and carefully tuning growth conditions. In fact, high-density QDs can be acquired quite easily by the Stranski-Krastanov (S-K) growth mode despite of random QDs’ nucleation and size distribution [7,8]. However, low-density QDs are relatively harder to acquire. Still several approaches have been developed to obtain low-density QDs structures by extremely low growth rate or precise control of the coverage close to the onset of two-dimensional (2D) to three-dimensional (3D) transition [9,10]. Additionally, some novel approaches such as modified droplet epitaxy [11,12] and pre-patterning by electron beam lithography combined with etching techniques [13,14] are also used to grow low-density QDs. Nevertheless, the growth conditions for low-density QDs structures are accordingly very different from those for high-density QDs structures. In this letter, we describe a growth method which demonstrates that the densities of InAs QDs on GaAs(001) substrates can be tuned in a wide range from 10^5^ to 10^10^ cm^−2^ by simply manipulating the V/III ratio via metal-organic chemical vapor deposition (MOCVD). This study provides a promising way to achieve reproducible and controllable growth of different QDs-based device structures by MOCVD.

## Methods

InAs QDs were grown on n-type GaAs(001) substrate via S-K growth mode by Thomas Swan/Aixtron low pressure MOCVD system (Aixtron SE, Herzogenrath, Germany). Trimethylindium (TMIn), trimethylgallium (TMGa), and arsine (AsH_3_) were used as the source materials with a carrier gas of H_2_. Prior to the InAs deposition, the substrate was heated to 750°C to remove the native oxides, and then a 500 nm thick GaAs buffer layer was grown at 620°C with V/III ratio of 50. Subsequently, the substrate temperature was lowered to 514°C for InAs QDs growth for 3.5 s. For all the samples studied, the only varied growth parameter was the flux of AsH_3_ flow. The flow rate of TMIn was fixed at 2.9 × 10^−4^ μmol·min^−1^, and the flow rates of AsH_3_ were varied from 0 to 0.29 μmol·min^−1^, which means that the V/III ratio was tuned from 0 to 1,000. During growth, the chamber pressure was kept at 150 mBar. After the deposition of the InAs QDs, the growth was interrupted for 30 s and then the substrate was cooled down to room temperature. The QD densities and morphologies were characterized by atomic force microscopy (AFM). For selected samples, 60 nm thick GaAs cap layers were deposited for the photoluminescence (PL) study.

## Results and discussion

**Figure 1:**
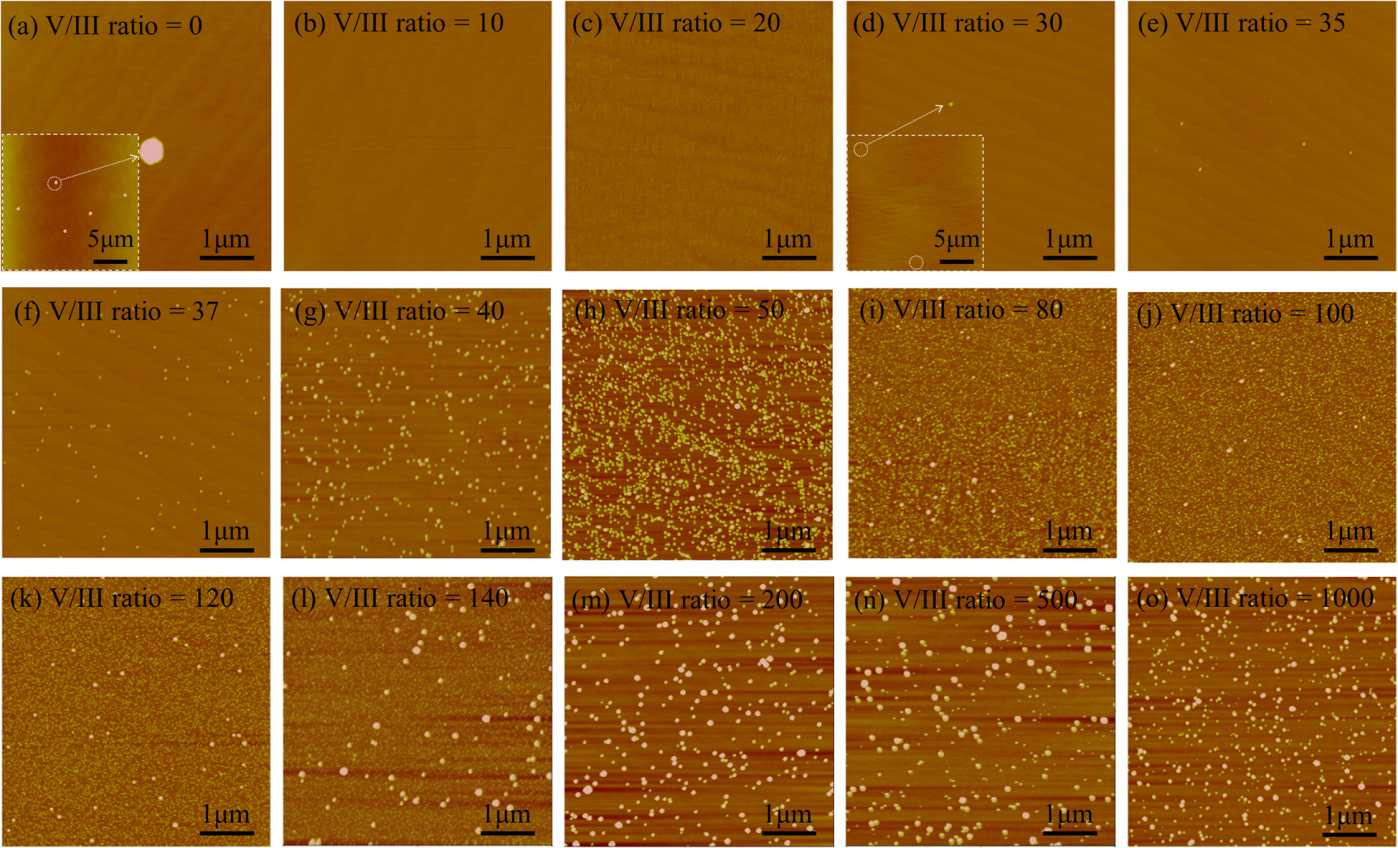
**AFM images of InAs quantum dots with different V/III ratios. (a-o)** AFM images of InAs quantum dots in a scan area of 5 μm × 5 μm with varied V/III ratios from 0 to 1,000. The inset figures in **(a)** and **(d)** are the corresponding AFM images of InAs QDs in a larger scan area of 20 μm × 20 μm.

**Figure 2:**
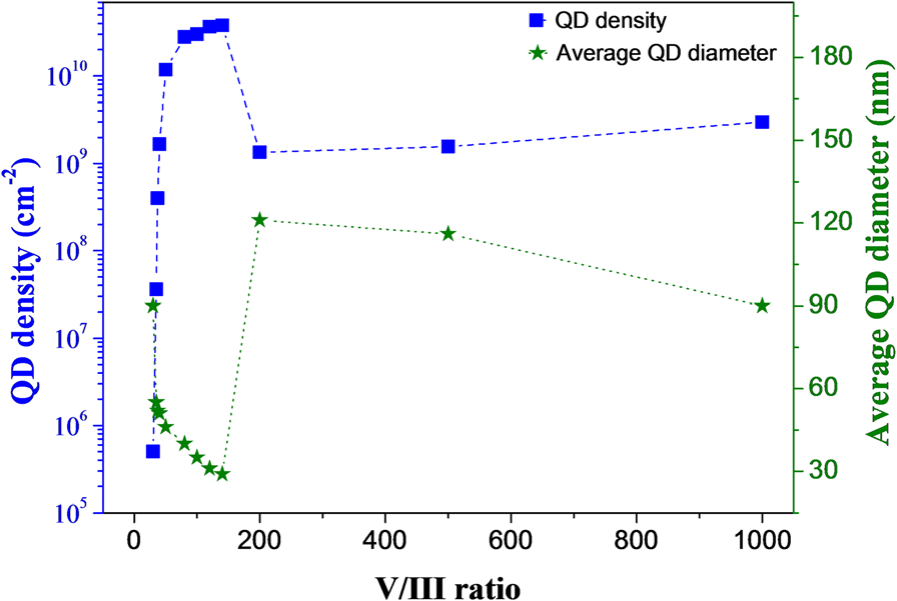
InAs QDs density and average base diameter as a function of V/III ratio.

AFM images of the InAs QDs deposited with varied V/III ratio are shown in Figure 1, and the corresponding densities and average base diameters as a function of V/III ratio are plotted in Figure 2, revealing strong effects of AsH_3_ partial pressure on the QDs formation. Large In droplets were formed at V/III ratio of 0 due to the absence of AsH_3_ molecules. After the introduction of AsH_3_, dramatic evolutions of InAs QDs are observed. From the AFM images corresponding to V/III ratio from 0 to 30, it is evident that the thickness of InAs layer at V/III ratio less than 30 is below the critical layer thickness with sample morphologies of flat surfaces. It also suggests that with V/III ratio at 30, the transition onset of growth mode from 2D to 3D occurs, and thus InAs QDs with ultra-low density (5 × 10^5^ cm^−2^) are acquired. Meanwhile, the relatively low AsH_3_ partial pressure (low V/III ratio) cannot limit the migration of In adatoms effectively; as a result, the InAs QDs have pretty large size with diameters around 90 nm.

We describe the detailed behavior of QDs with increasing V/III ratio by three phases as follows:

*Phase I*. Small increments of AsH_3_ partial pressure by increasing V/III ratio to 35, 37, 40, and 50 result in rapid increases of well-developed QDs. The QD density increases nearly by five orders of magnitude, from 5 × 10^5^ cm^−2^ (V/III ratio = 30) to 1.2 × 10^10^ cm^−2^ (V/III ratio = 50). Also, the base diameters decrease correspondingly from 90 to 46 nm.

*Phase II*. By further increasing the V/III ratio from 50 to 140, the densities of QDs increase slowly from 1.2 × 10^10^ cm^−2^ to 3.8 × 10^10^ cm^−2^, and the corresponding base diameters decrease from 46 to 29 nm. Also, we notice that the uniformity of QDs gets worse and the bimodal size distribution of QDs gets more obvious with increasing V/III ratio.

*Phase III*. The density of QDs decreases significantly by one order of magnitude when the V/III ratio is increased up to 200, and then increases slowly again with higher V/III ratio. During this phase, the average base diameters also undergo abrupt change, increasing to 121 nm and then decreasing to 90 nm.

To explain the above complicated behaviors of QDs, several competing mechanisms should be taken into account. Phase I is in the margin of 2D to 3D transition which is reasonable to conclude from the AFM images; therefore, a minor increase of coverage can facilitate the growth changing from 2D to 3D, thus resulting in significant change of QDs. As the AsH_3_ partial pressure increases, the rate of the chemical reaction of TMIn+AsH_3_→InAs+3CH_4_ is increased by providing more available AsH_3_ molecules, leading to the increasing coverage of InAs. As a result, the QD density increases dramatically. A similar behavior of increasing dot density with increasing coverage can be found in many other reports [9,15,16]. Meanwhile, the increased AsH_3_ partial pressure can limit the migration length of In adatoms; therefore, the base diameter tends to decrease. Accordingly, in phase I, with the increasing of V/III ratio, the QD densities increase dramatically and the corresponding QD average diameters decrease.

In phase II, the chemical reaction rate as well as the InAs coverage keeps increasing due to the increasing AsH_3_ partial pressure, but the increase of the growth rate is limited by the fixed TMIn flow rate. Furthermore, phase II is beyond the 2D to 3D transition; therefore, the QD density increases with decreasing rate. Similarly, the average base diameters decrease due to the limited In migration length with increasing AsH_3_ partial pressure. In addition, considering the kinetics of MOCVD growth, the initial formation of QDs is not in the thermal equilibrium; thus, increasing coverage also leads to the development of small QDs into energetically favorable large-sized QDs. In our case, the bimodal size distribution starts occurring at V/III ratio of 50 and gets more obvious with increasing V/III ratio.

In phase III, the QD density decreases significantly at V/III ratio of 200. Apparently, a different mechanism may play a dominant role. At high V/III ratio, the available AsH_3_ molecules are far more than enough for group III species, thus the excess AsH_3_ may act as impurity-free ‘morphactants’ and raise the surface energy [17], leading to the suppression of QD formation. This effect becomes prominent with the increase of V/III ratio, finally causing the sudden decrease of QD density at V/III ratio of 200 (phase III). However, with further increase of V/III ratio, the QD density increases gently. The reasons are still not clear at this moment, but in this case, the partial pressure of group III species becomes so low that the possibility of surface reconstruction, which is not detectable during MOCVD growth, may need to be considered. Further experimental works will be conducted to clarify this phenomenon.

**Figure 3:**
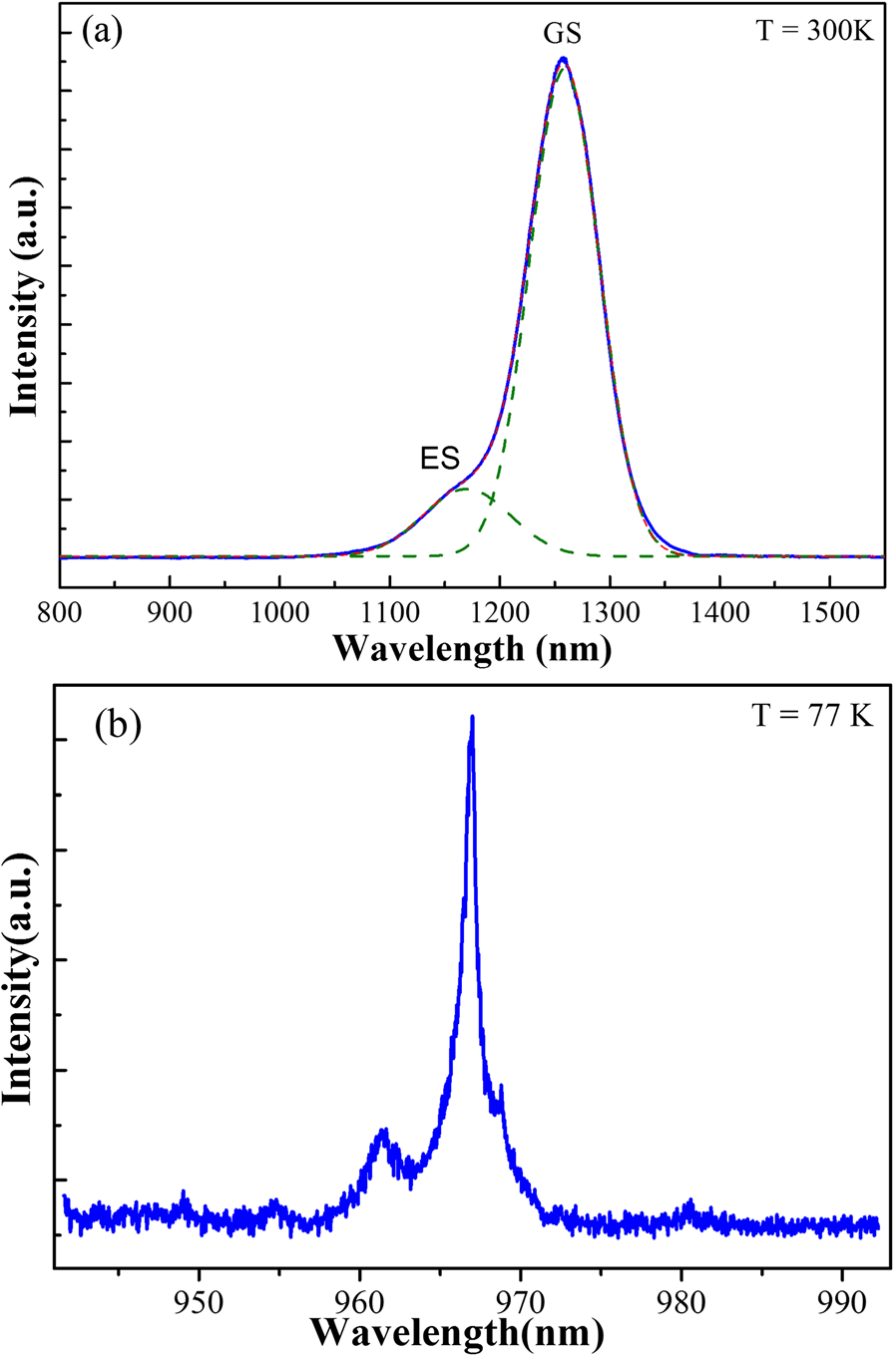
**Results of PL measurements of selected samples. (a)** Room-temperature PL spectrum of GaAs/InAs QD (V/III ratio = 50)/60 nm GaAs cap measured at 300 K. **(b)** The μPL spectrum of GaAs/InAs QD (V/III ratio = 35)/60 nm GaAs cap measured at 77 K.

The PL measurements of selected samples were conducted and the results are shown in Figure 3. Figure 3a shows the photoluminescence from an ensemble of GaAs/InAs QD (V/III ratio = 50)/60 nm GaAs cap measured at 300 K using excitation at 514 nm. The ground state (labeled as GS) emission peak and the excited state (labeled as ES) emission peak are identified by fitting the PL spectra with two Gaussians. The full width at half maximum of the GS emission peak is 63 nm, indicating that the uniformity of the QDs should be further improved by optimizing other growth parameters. Low-temperature (77 K) μPL using excitation at 514 nm was measured for the ensemble of GaAs/InAs QD (V/III ratio = 35)/60 nm GaAs cap (Figure 3b). The emission peak at 966.8 nm indicates that the ensemble has single QD emission characteristics, suggesting that this growth approach can be used for the fabrication of single-photon devices.

## Conclusions

In conclusion, we have described the effects of the V/III ratio on the density and sizes of InAs QDs. Due to the effects of several competing mechanisms resulting from increasing AsH_3_ partial pressure on coverage, In adatom migration length and surface energy, which are the complicated behaviors of QD formation, are observed. The results also demonstrate that the densities of InAs QDs can be manipulated easily in a wide range from 10^5^ to 10^10^ cm^−2^ by varying the V/III ratio. Although the initial PL studies show that the optical performance of InAs QDs should be further improved, this V/III ratio-dependent InAs QDs growth approach may prove very useful for the MOCVD growth of different QDs-based device structures due to its simplicity and reproducibility.

## Abbreviations

2D: Two-dimensional; 3D: Three-dimensional; AFM: Atomic force microscopy; AsH3: Arsine; ES: Excited state; GS: Ground state; MOCVD: Metal-organic chemical vapor deposition; PL: Photoluminescence; QDs: Quantum dots; S-K: Stranski-Krastanov; TMIn: Trimethylindium; TMGa: Trimethylgallium.

## Competing interests

The authors declare that they have no competing interests.

## Authors’ contributions

SLL wrote the manuscript and participated in all the experiments and the data analysis. QQC and SCS participated in all the experiments and the data analysis. YLL, QZZ, JTL, XHW, JBH, and JPZ took part in the discussions and testing of PL. CQC and YYF supervised the writing of the manuscript and all the experiments. All authors read and approved the final manuscript.

## Authors’ information

LSL, YLL, and JPZ are PhD students at Huazhong University of Science and Technology. QQC and SCS are Master’s degree students at Huazhong University of Science and Technology. JBH and CQC hold professor positions at Huazhong University of Science and Technology. YYF holds an associate professor position at Huazhong University of Science and Technology. QZZ is a PhD student at Sun Yat-Sen University. JTL and XHW hold professor positions at Sun Yat-Sen University.
